# Novel TBC1D24 Mutations in a Case of Nonconvulsive Status Epilepticus

**DOI:** 10.3389/fneur.2018.00623

**Published:** 2018-07-31

**Authors:** Jingjing Li, Ruihong Liu, Huiyu Feng, Jian Zhang, Dilong Wang, Yiming Wang, Jinsheng Zeng, Yuhua Fan

**Affiliations:** ^1^Department of Neurology and Stroke Center, First Affiliated Hospital, Sun Yat-sen University, Guangzhou, China; ^2^Fifth Affiliated Hospital, Sun Yat-sen University-BGI Laboratory, Department of Experimental Medicine, Fifth Affiliated Hospital, Sun Yat-sen University, Zhuhai, China; ^3^Xinhua College, Sun Yat-sen University, Guangzhou, China

**Keywords:** nonconvulsive status epilepticus, TBC1D24, cerebellar ataxia, mutation, novel mutation

## Abstract

**Objective:** Nonconvulsive status epilepticus (NCSE) is an uncommon clinical manifestation in patients with TBC1D24 mutations. In addition, NCSE has not been reported as a syndrome together with cerebellar ataxia and ophthalmoplegia.

**Methods:** We herein report the clinical and genetic features of a four-year-old patient with NCSE, cerebellar ataxia, and ophthalmoplegia caused by hitherto unidentified TBC1D24 mutations. We performed 24-h video electroencephalogram (EEG), magnetic resonance imaging, and gene sequencing on the patient and her parents to determine the diagnosis.

**Results:** We identified a novel c.1416_1437del (p.Ser473Argfs^*^43) mutation, as well as the previously identified c.1499C>T (p.Ala500Val) mutation in TBC1D24, by using targeted next-generation sequencing. The novel mutation (inherited from the mother) is the first reported deletion mutation longer than 20 bp in TBC1D24. The p.Ala500Val mutation inherited from father has been reported in a German patient with infantile myoclonic, for whom results from the EEG and neuroimaging were normal. These two mutations resulted in the severe phenotypes observed in our patient

**Conclusions:** The identification of the novel TBC1D24 mutation and consequent complicated clinical manifestations suggest that patients with NCSE and ataxia demand more attention. We further recommend that genetic test should be administered to these patients to avoid genetic inheritance of this mutation.

## Introduction

According to a consensus of the International League Against Epilepsy (ILAE), status epilepticus (SE) is defined by either the failure to terminate seizures, or the initiation of mechanisms that cause seizures of an abnormally long duration (>5 min).(1) Approximately 20–25% of SE cases are nonconvulsive (2, 3); nonconvulsive status epilepticus (NCSE) is characterized by a seizure-induced prolonged change in a person's level of consciousness without motor manifestations (2). The *TBC1D24* (*TBC1 domain family member 24*) gene encodes a member of the Tre2-Bub2-Cdc16 (TBC) domain-containing RAB-specific GTPase-activating proteins, which coordinate Rab proteins and other GTPases for the proper transport of intracellular vesicles. The *TBC1D24* also contains a TBC lysine motif catalytic (TLDc) domain involved in oxidative stress resistance. A mutation of *TBC1D24* was confirmed to relate to epilepsy (EP) or EP syndrome. The clinical symptoms of *TBC1D24-*related epilepsy include focal epilepsy, dysarthria, mild to moderate intellectual disability, and familial infantile myoclonic epilepsy. As previously reported, different mutations in *TBC1D24* cause various epileptic syndromes.(4–8) We herein report a previously unidentified mutation of the *TBC1D24* gene in a patient who presented with NCSE, cerebellar ataxia and ophthalmoplegia with novel mutation of *TBC1D24* gene.

## Case presentation

### Subjects

**Figure 1:**
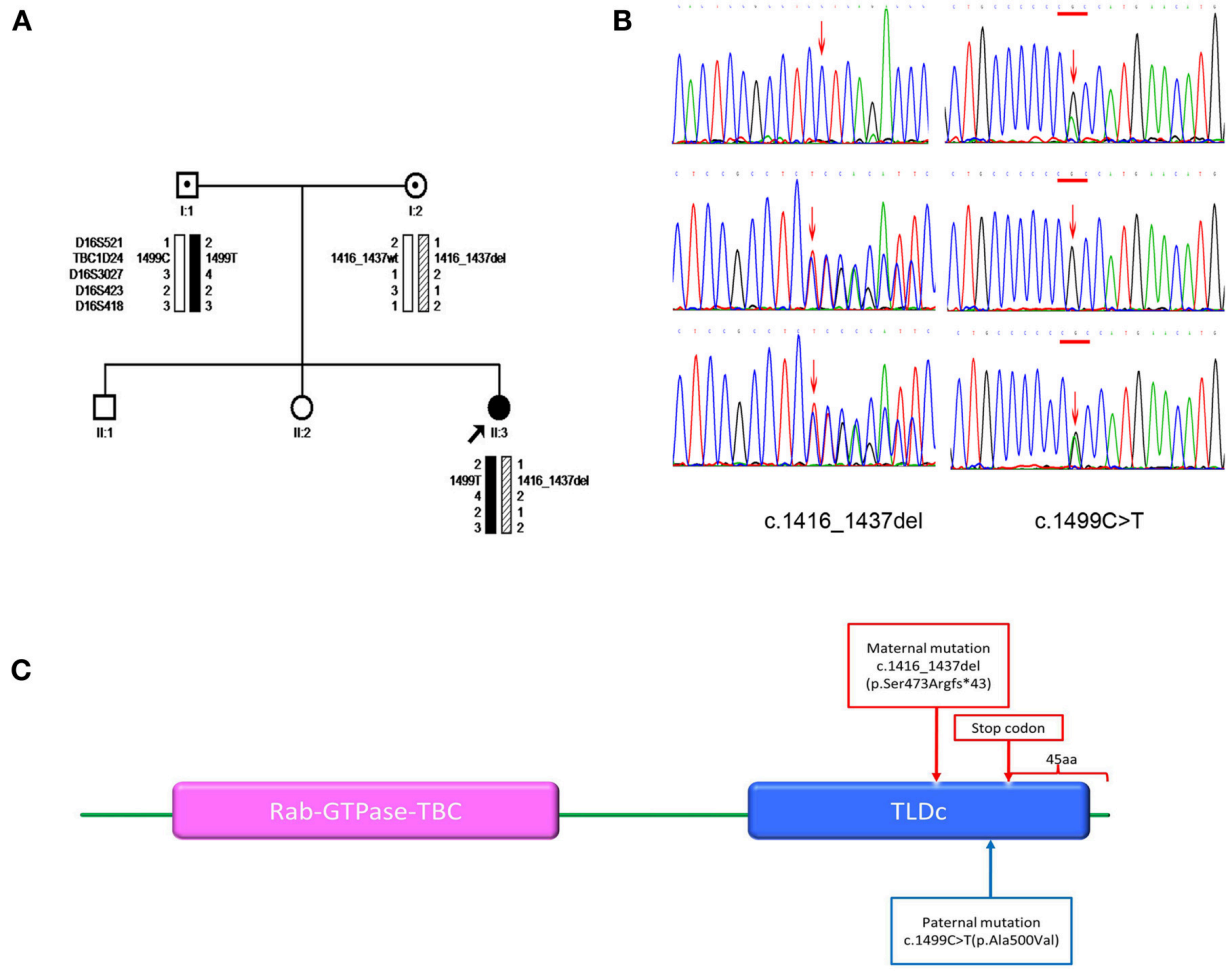
**(A)** Pedigrees for the family and haplotype construction. Filled circles indicate patient. Arrows point to the probands for the family. Haplotypes are displayed below the symbols of the family members available for analysis. Markers used in the haplotype construction are listed on the left. Black bars depict the paternal disease haplotype; hatched bars, the disease haplotype of the mother; and open bars, the haplotypes that do not contain a variant. Wt denotes the wild-type allele. **(B)** Sanger sequencing. The members of the family are listed on the left. Left-pointing arrows denote the forward sequence of c.1416_1437del (p.Ser473Argfs*43) in TBC1D24. Right-pointing arrows indicate the forward sequence of c.1499C>T (p.Ala500Val) in TBC1D24. Arrow indicates the locations of the mutations. **(C)** Domain information for the protein encoded by the *TBC1D24* gene. Arrows indicate the locations of the mutation (p.Ser473Argfs*43) and the premature stop codon introduced by the deletion mutation. The wild-type TBC1D24 protein has 559 amino acids. It contains both the Rab GTPase domain (purple) and TLDc domain (blue). The mutation p.Ser473Argfs*43 is predicted to delete 45 amino acids from the C terminus of the TBC1D24 protein.

The present study involved one patient (II-3), her two siblings (II-1 and II-2), and their unaffected parents (I-1 and I-2) from a nonconsanguineous marriage (Figure 1A). DNA was extracted from the peripheral blood of available family members (Figure 1A) using the QIAamp DNA Kit (Qiagen, Germany). All participants gave written informed consent. The study was approved by the Ethics Committee of the Sun Yat-sen University for Human Study and was conducted in accordance with the principles of the 1975 Declaration of Helsinki. Results from clinical manifestation, 24-h-video electroencephalogram (EEG), magnetic resonance imaging (MRI), and lab tests were analyzed.

### Genetic analysis

We aimed to identify damaging segments of the patient's DNA: single nucleotide variations (SNVs); small insertions and deletions (indels) that cause non-synonymous, frameshift, inframe changes; and variants that occurred at splice sites. A targeted next-generation sequencing was thus performed on the patient's genomic DNA using the TruSight One Sequencing Panel (Illumina Inc, USA), which contains 700 genes suspected of having a role in epilepsy.

We used Sanger sequencing to confirm the mutations identified on the proband. We also screened the mutations of *TBC1D24* (gene ID: 57465) in parents. To determine possible effects of the mutations, we used SIFT (http://sift.jcvi.org/), PolyPhen2 (http://genetics.bwh.harvard.edu/pph2/) and Mutation Taster (http://www.mutationtaster.org/). We classified mutations using the Guidelines of the American College of Medical Genetics and Genomics (ACMG) (9). For single nucleotide variants, CLUSTAL X (1.81) was used to compare the human TBC1D24 amino acid sequence (Homo sapiens NP_001186036.1) with five orthologues and predict the evolutionary conservation across species (rhesus monkey (*Macaca mulatta*), NP_001244494.1; cattle (*Bos taurus*), NP_001039761.1; rat (*Rattus norvegicus*), NP_001099239.1; mouse (*Mus musculus*), NP_001157319.1; chicken (*Gallus gallus*) NP_001244200.1). The National Center for Biotechnology Information Open Reading Frame Finder (ORF Finder, http://www.ncbi.nlm.nih.gov/gorf/gorf.html) was used to predict consequences of the frame shifting mutations. Domain and motif information for the encoded wild proteins was obtained from Uniprot (http://www.uniprot.org/uniprot/Q9ULP9). We also performed haplotype analyses for the regions spanning *TBC1D24* with microsatellite markers (D16S521, D16S3027, D16S423, and D16S418; Applied Biosystems PRISM Linkage Mapping Set Version 2.5, www.appliedbiosystems.com). Haplotypes were constructed using the Cyrillic 2.1 (http://www.cyrillicsoftware.com/).

### Clinical findings

A four-year-old girl was admitted to our department complaining of developmental delay and a sudden onset of diminished vision. She had a 4-year history of two types of paroxysmal symptoms: (1) generalized tonic-clonic seizure; (2) rhythmic unilateral facial spasms observed 3 months after birth, which were accompanied by fixation of the eyes to one side. She was diagnosed with “epilepsy” at a local hospital (at the age of 3 years) and was thenceforth administered antiepileptic medication. The patient exhibited spasms in the craniofacial region and all of her limbs, as well as a concurrent, sudden decrease in vision manifested as lethargy and constant crying. The patient had ocular hypertelorism. Before the patient was 2 years old, she could not finish the activity such as sit, walk and jump as the others in the same age. She could walk steadily only when she was 3 years old and she rarely exhibited spontaneous speech and could only verbalize her name and age at 4 years old.

Her left eye was in the abduction position since she was about 1 year old. It is not a transient manifestation and it didn't recover after the other symptoms such as diminished vision and sustaining crying recovered. Other cranial nerve examinations revealed no further abnormalities. Ophthalmologic examinations yielded no indication of amblyopia, lateral rectus palsy, or other ophthalmologic problems. Her vision and visual field were normal.

**Figure 2:**
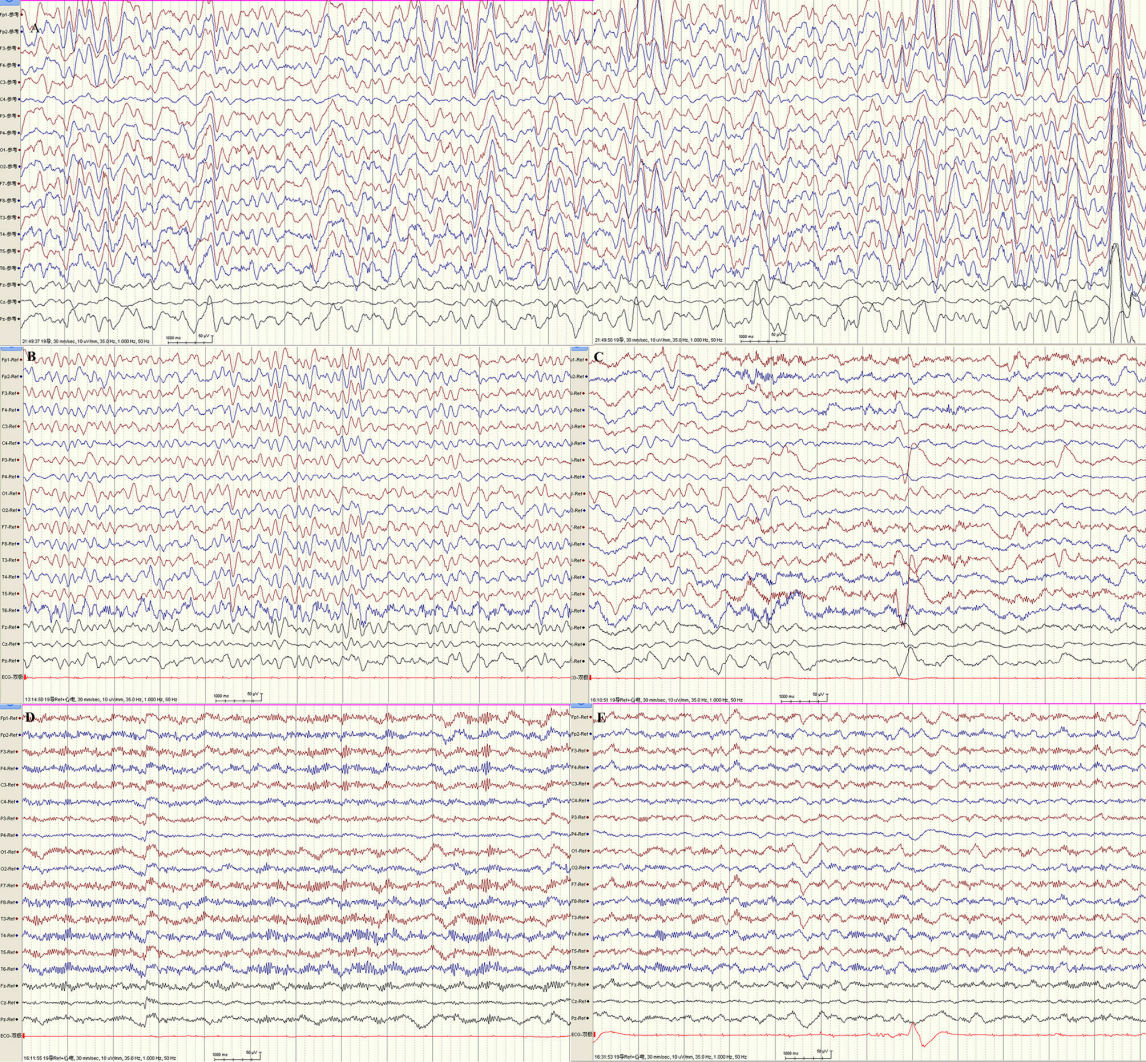
24 h-EEG monitoring indicated non-convulsive status epilepticus. **(A,B)** Predominant theta and delta activity in bilateral hemispheres. With the increase in voltage from 30 to 485 μV, the frequency decreased from 6 to 2.5 Hz. **(C)** The frequency of beta activity increased while theta activity decreased 1 min following intravenous injection of diazepam. **(D)** Beta activity were bilaterally predominant nearly 1 hr following injection. **(E)** At about 1.5 h after intravenous injection of diazepam, the EEG recovered to normal background (majority beta activity with maximum amplitudes ranged from 18.5 to 30.5 μV at frequencies of 5–30 Hz, scattered theta with maximum amplitudes ranged from 20 to 40 μV at frequencies of 5–7.5 Hz). By this time, the patient's crying subsided, her vision recovered, and she could answer questions as usual.

EEG monitoring conducted over the course of 24 h found predominant theta and delta activity in bilateral hemispheres. With the increase in voltage from 30 to 485 μV, the frequency decreased from 6 to 2.5 Hz. (Figures 2A,B). The phenomenon acutely abolished after intravenous injection of diazepam and frequency of beta activity increased and finally recovered to normal EEG background (Figure 2D). The EEG of our patient is in accordance with the 4a EEG criteria of NCSE by Kaplan (10). In addition, the clinical symptoms recovered. The patient's crying subsided, her vision recovered and she was able to answer questions as usual. Our legend of Figure 2 further includes all relevant details.

**Figure 3:**
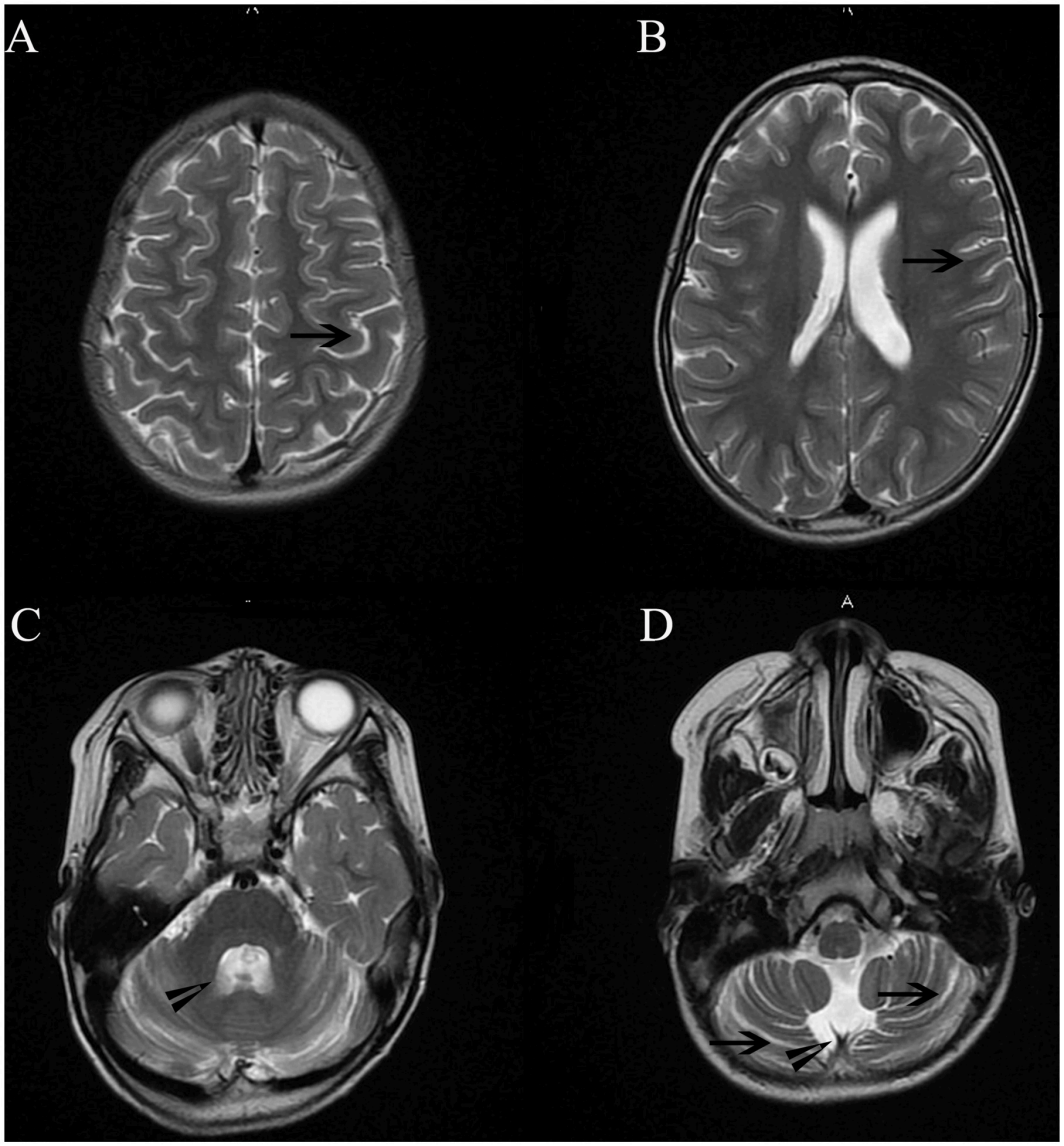
Cranial MRI showed mild atrophic changes in bilateral hemisphere and cerebellum. Ventricular systems were generally enlarged. Cerebral sulcus and fissures widened in bilateral hemisphere and cerebellum. Long arrowheads in Figure **(A,B,D)** showed widened sulcus and fissures in hemisphere and cerebellum. Short arrowheads in **(B,C)** showed enlarged the fourth ventricle and cisterna magna.

Cranial MRI was performed using a 3.0-T magnetic resonance scanner (MAGNETOM Trio Tim, Siemens Healthcare, Erlangen, Germany). Conventional T1-weighted (T1WI), T2-weighted (T2WI), and fluid-attenuated inversion recovery (FLAIR) were obtained. Analysis of the imaging revealed mild atrophic changes in both hemispheres, as well as the cerebellum (Figure 3). Ventricular systems were generally enlarged. Cerebral sulcus and fissures widened in bilateral hemisphere and cerebellum.

The patient's gait was wide-based and unsteady. She could not complete the Romberg test with her eyes closed. Bilateral coordinated movement could not be successfully performed. Together with the mild atrophic cerebellum in MR imaging, these manifestations suggest cerebella ataxia.(Figure 3) The patient's parents could not remember the exact time point when her left eye was in abduction position. There was no proof of ophthalmological problems such as amblyopia or lateral rectus palsy. In addition, no lesions were found in her brainstem and CSF analysis were normal.

The cause of ophthalmoplegia is thus uncertain. The fact that this symptom has rarely been reported in patient with TBC1D24 mutations further confounds our analysis. It may, however, relate to the novel TBC1D24 mutation. Future research is needed to confirm this. Her parents' neurological examination results were normal.

### Mutation analysis

We identified and confirmed two compound heterozygous mutations in TBC1D24 (NM_001199107.1) by using next-generation sequencing and Sanger sequencing, respectively: c.1416_1437del (p.Ser473Argfs^*^43) and c.1499C>T (p.Ala500Val; Figure 1B). The former mutation is novel, whereas the latter has been reported in a patient with infantile myoclonic epilepsy (11). Sequencing and haplotype construction of the parental DNA revealed that the mother was the heterozygous carrier of p.Ser473Argfs^*^43, and the father of p.Ala500Val. The p.Ser473Argfs^*^43 mutation occurred on exon 6, causing a frameshift. It is further predicted to introduce a premature stop codon at position 514, thereby truncating 45 amino acids at the C terminus of the TBC1D24 protein (ORF Finder predicted; Figure 1C). Mutation Taster classified the anomaly as “disease causing” that may induce nonsense mediated decay (NMD). The p.Ser473Argfs^*^43 mutation truncated the TLDc domain of the TBC1D24 protein, while p.Ala500Val was found to occur in the same region; both mutations were classified as “pathogenic” according to ACMG guidelines. PolyPhen2 classified the mutation as “probably damaging,” and SIFT predicted it as “damaging.” Across-species alignment of wild type TBC1D24 protein demonstrated that the p.Ala500 is highly conserved in humans, rhesus monkeys, cattle, rats, mice, and chickens.

## Discussion

We diagnosed a novel TBC1D24 mutation, c.1416_1437del (p.Ser473Argfs^*^43), in a four-year-old girl who presented with NSCE on admission. To the best of our knowledge, this mutation constitutes the first reported TBC1D24 deletion mutation longer than 20 bp. Her clinical manifestations included ocular hypertelorism, developmental delay, onset of NCSE, and a wide-based, unsteady gait. Genetic screening for EP-related genes revealed a novel mutation inherited from her mother, in whom the TBC1D24 mutation was identified in the verified mutant site. The TBC1D24 mutation was confirmed to relate to EP or EP syndrome. Corresponding epilepsy syndromes exhibit marked phenotypic pleiotropy, with a severity spectrum ranging from isolated deafness, benign myoclonic epilepsy restricted to childhood, and normal intellect, to early-onset epileptic encephalopathy, severe developmental delay, and early death.(11–13) The disorder features similarities with a other types of epilepsy, including familial infantile myoclonic epilepsy, early infantile epileptic encephalopathy 16; and a number of conditions other than epilepsy such as Doors syndrome (deafness, onychodystrophy, osteodystrophy, mental retardation, and seizures), autosomal recessive deafness 86, and autosomal dominant deafness 65 (OMIM:www.omim.org).

The clinical presentation of our case featured developmental delay, NCSE, ataxia, and left eye in the abduction position. Though these symptoms have been associated individually with TBC1D24-related epilepsy syndromes, our report is—to the best of our knowledge—the first to present NCSE, cerebellar ataxia, and ophthalmoplegia together as a syndrome.

The patient's left eye adopted the abduction position at about 1 year old. The patient's parents could not remember the exact time point. It was a persistent phenomenon and there was no evidence of other ophthalmologic problems, such as amblyopia or a lateral rectus palsy. Furthermore, no lesions were found in brain stem, and analysis of the CSF revealed no abnormalities. Ophthalmoplegia is a clinical symptom and the cause of ophthalmoplegia was uncertain, and this symptom has rarely been reported in patient with any TBC1D24 mutation. The symptom may, however, relate to the novel TBC1D24 mutation identified by this study. Future research is needed to confirm this association. As mentioned in the clinical finding, the EEG and clinical presentation was in accordance with the criteria of NCSE (10), and we can exclude the toxic, metabolic, endocrine causes based on the history,manifestation and lab test.

The p.Ala500Val mutation inherited from the father was reported in a German patient who also exhibited the p.Phe229Ser mutation in TBC1D24; EEG and neuroimaging were performed and yielded normal results. Compounded with the novel maternal truncated mutation, the paternal mutation resulted in severe phenotypes in our patient. To the best of our knowledge, most of the mutations in TBC1D24 are located in the TBC domain, while only four are reported in TLDc domain (14). Our patient is the first case reported to feature compound heterozygous mutations located in TLDc domain. However, the function of the TLDc domain in TBC1D24 is largely unknown (15). This case report therefore not only expands the TBC1D24 mutation spectrum, but also helps to explore the pathogenesis of mutations in TLDc domain of TBC1D24. Furthermore, the pathogenic mutations identified by this study may aid in prenatal diagnoses; our findings serve as a reminder of the potential complications of NCSE and ataxia, and justify the administration of genetic tests to these patients to avoid genetic inheritance of this disorder.

## Patient consent

All participants gave written informed consent. Since the patient was only 4 years-old, her parents signed the consent form and allowed us to publish this case report.

## Ethics statement

This study was carried out in accordance with the recommendations of the committee of the first affiliated hospital of Sun Yat-sen university. The protocol was approved by the ethics committee of the first affiliated hospital of Sun Yat-sen university on human research. All subjects gave written informed consent in accordance with the Declaration of Helsinki.

## Author statement

This study was conducted by all aforementioned authors. All participants gave written informed consent. They have authorized JL to submit or publish the work on their behalf. The authors thank the patient and her family for participating in this study.

## Author contributions

JL, RL, and YF design of the study, analysis of the data, drafting of the manuscript. JSZ and YW revision of the manuscript for intellectual content. HF, JZ, and DW collection and analysis of data.

### Conflict of interest statement

The authors declare that the research was conducted in the absence of any commercial or financial relationships that could be construed as a potential conflict of interest. The reviewer ZW and handling Editor declared their shared affiliation at the time of the review.
